# Dominant, toxic gain-of-function mutations in *gars* lead to non-cell autonomous neuropathology

**DOI:** 10.1093/hmg/ddv176

**Published:** 2015-05-13

**Authors:** Stuart J. Grice, James N. Sleigh, William W. Motley, Ji-Long Liu, Robert W. Burgess, Kevin Talbot, M. Zameel Cader

**Affiliations:** 1 MRC Functional Genomics Unit, Department of Physiology, Anatomy and Genetics, University of Oxford, South Parks Road, Oxford OX1 3PT, UK,; 2 Nuffield Department of Clinical Neurosciences, University of Oxford, John Radcliffe Hospital, Oxford OX3 9DU, UK,; 3 The Weatherall Institute of Molecular Medicine, University of Oxford, John Radcliffe Hospital, Oxford OX3 9DS, UK,; 4 Neurogenetics Branch, National Institute of Neurological Disorders and Stroke, NIH, Bethesda, MD 20892, USA and; 5 The Jackson Laboratory, Bar Harbor, ME 04609, USA

## Abstract

Charcot–Marie–Tooth (CMT) neuropathies are collectively the most common hereditary neurological condition and a major health burden for society. Dominant mutations in the gene *GARS*, encoding the ubiquitous enzyme, glycyl-tRNA synthetase (GlyRS), cause peripheral nerve degeneration and lead to CMT disease type 2D. This genetic disorder exemplifies a recurring motif in neurodegeneration, whereby mutations in essential, widely expressed genes have selective deleterious consequences for the nervous system. Here, using novel *Drosophila* models, we show a potential solution to this phenomenon. Ubiquitous expression of mutant GlyRS leads to motor deficits, progressive neuromuscular junction (NMJ) denervation and pre-synaptic build-up of mutant GlyRS. Intriguingly, neuronal toxicity is, at least in part, non-cell autonomous, as expression of mutant GlyRS in mesoderm or muscle alone results in similar pathology. This mutant GlyRS toxic gain-of-function, which is WHEP domain-dependent, coincides with abnormal NMJ assembly, leading to synaptic degeneration, and, ultimately, reduced viability. Our findings suggest that mutant GlyRS gains access to ectopic sub-compartments of the motor neuron, providing a possible explanation for the selective neuropathology caused by mutations in a widely expressed gene.

## Introduction

Mutations in several aminoacyl-tRNA synthetase (ARS) genes have been linked to different forms of Charcot–Marie–Tooth (CMT) disease (1–6), a heterogeneous set of conditions characterized by progressive distal muscle wasting, weakness and sensory dysfunction (7). Dominant mutations in *GARS* (ENSG00000106105) cause CMT type 2D (CMT2D, OMIM ID 601472) (1). *GARS* encodes the non-redundant, homodimeric enzyme, glycyl-tRNA synthetase (GlyRS), which covalently links the amino acid glycine to its cognate tRNA, making it essential for protein translation fidelity. Two *GARS* translational start sites result in the production of mitochondrial and cytoplasmic GlyRS isoforms. GlyRS possesses three functional domains common to both isoforms: a highly conserved N-terminal WHEP-TRS domain of unclear function, a catalytic core, and a C-terminal anticodon-binding domain.

Two mouse models of CMT2D, *Gars^Nmf249/+^* and *Gars^C201R/+^*, result from dominant amino acid substitutions and display features akin to the human condition, including muscle weakness and peripheral axon degeneration (8,9). Mouse phenotypes do not correlate with aminoacylation function (8–11), a 50% reduction in *Gars* mRNA levels results in no overt phenotype (8), and *GARS* mutations have differential effects on GlyRS dimerization (11); however, defects in the canonical function of GlyRS have not been completely ruled out as a possible contributing factor in the human disease (12). Interestingly, neuromuscular junction (NMJ) maturation defects precede lower motor neuron connectivity abnormalities in mice, indicating that defective synapse development may play a role in pathology (13). Furthermore, overexpression of wild-type human *GARS* does not rescue CMT2D mice, suggesting a toxic gain-of-function of mutant GlyRS as the disease trigger (14). Consistent with this, a number of CMT2D-associated *GARS* mutations have been shown to cause the same conformational opening of the protein thereby exposing a consensus neomorphic area with potential for aberrant protein interactions (15).

The *Drosophila melanogaster gars* gene (also known as *aats-gly*) shares 60% homology with human *GARS*, and most patient mutations affect amino acids conserved in the fly (16). Using mosaic analysis with a repressible cell marker, homozygous null *gars* mutations were shown to affect branching of olfactory bulb and mushroom body neurons (17). However, this recessive loss-of-function model does not accurately reflect human CMT—a dominant disorder where mutant *GARS* is expressed in all tissues. Subsequent experiments in *Drosophila* have specifically looked at cell autonomous neuronal toxicity of mutant GlyRS (18). We now describe a new *Drosophila* model expressing a dominant disease allele that we use to identify novel features of the GlyRS toxic gain-of-function mechanism, and in particular inter-cell type interactions, which may contribute to the pathology of CMT2D.

## Results

### Non-neuronal *gars^P234KY^* expression leads to motor deficits and reduced lifespan

All CMT2D-associated mutations are located downstream of the 54 amino acid mitochondrial targeting sequence, clustering at the dimer interface (11,15,19,20). We therefore created *UAS* constructs to express *Drosophila* wild-type and mutant cytoplasmic, not mitochondrial, *gars*, focusing on the *P234KY* mutation—the equivalent *Drosophila* and human mutations corresponding to *P278KY* in the *Gars^Nmf249/+^* mouse. This residue is in ‘kissing contact’ with residues I280 and C201 mutated in human disease and the second *gars* mouse model, respectively (15).

**Figure 1.**
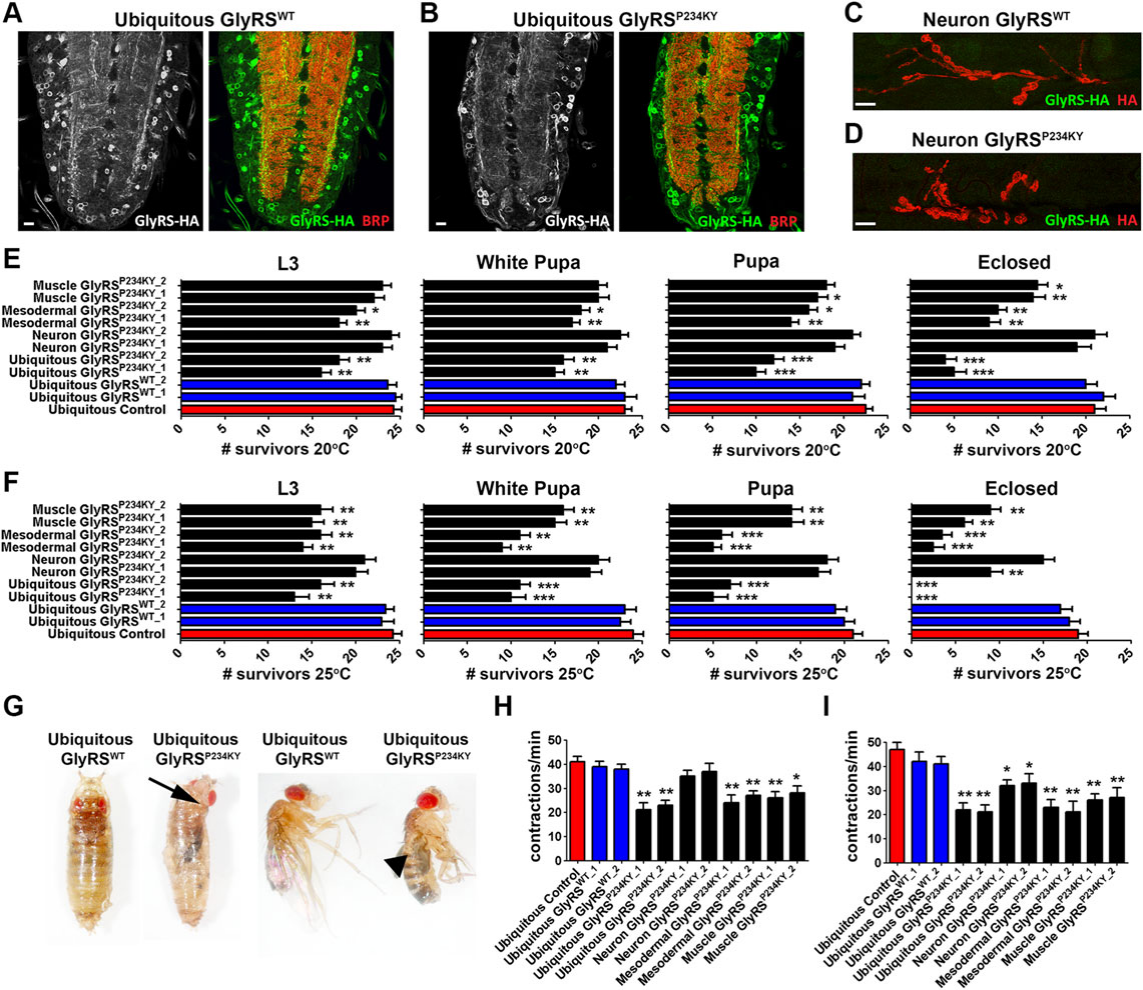
Dominant, toxic effects caused by GlyRS^P234KY^ lead to non-cell autonomous longevity and motor defects. (**A**) GlyRS^WT^ and (**B**) GlyRS^P234KY^ localization in the central nervous system of larvae ubiquitously (*1032-GAL4*) expressing *UAS-gars^WT_2^* and *UAS-gars^P234KY_2^*, respectively. (**C**) GlyRS^WT^ and (**D**) GlyRS^P234KY^ localization at the NMJ in larvae neuronally expressing *UAS-gars^WT_2^* and *UAS-gars^P234KY_2^*, respectively. Note that GlyRS expressed in neurons does not reach the peripheral synapse. (**E** and **F**) GlyRS^P234KY^ toxicity was analysed by expressing *gars^P234KY^* ubiquitously (*1032-GAL4*), in mesoderm (*how-GAL4*), muscle (*MHC-GAL4*) or neurons (*elav-GAL4*) at 20°C (E) and 25°C (F). Transgene expression is lower at 20 than 25°C. Flies ubiquitously expressing *gars^WT^* (blue bars) and *CD8::GFP* (red bars) were used as controls. (**G**) Mutant *gars^P234KY^* flies reach the pupal stage, but fail to eclose and often become trapped in their pupal cases (arrow). The few flies that escape and reach adulthood display wing expansion defects (arrow head). (**H**) Ubiquitous, mesodermal and muscle expression of *gars^P234KY^* caused a significant loss of muscle contractions at 20°C. (**I**) Ubiquitous, mesodermal, muscle and neuronal driven *gars^P234KY^* expression also reduced larval muscle contractions at 25°C. For all graphs, *P < 0.05; **P < 0.01; ***P < 0.001 Dunn's multiple comparison test. For survival studies, 25 flies per genotype were scored over four independent experiments. For muscle contraction studies, at least 15 larvae were analysed per genotype. Scale bars = 10 μm.

To mimic the dominant *GARS* mutations that cause CMT2D, and to better understand the selective neuronal pathology seen in the disease, we expressed the cytoplasmic isoform of wild-type (*UAS-gars^WT^*, which produces GlyRS^WT^) and mutant (*UAS-gars^P234KY^*, which produces GlyRS^P234KY^) *Drosophila gars* transgenes using the UAS-GAL4 system (18). Two different constructs were used for both the wild-type (*UAS-gars^WT_1^* and *UAS-gars^WT_2^*) and the mutant (*UAS-gars^P234KY_1^* and *UAS-gars^P234KY_2^*) transgenes, with *UAS-gars^WT_1^* and *UAS-gars^P234KY_1^* being the same as those used by Ermanoska *et al*. (18), and *UAS-gars^WT_2^* and *UAS-gars^P234KY_2^* being novel to this work. *UAS-gars^WT_1^* and *UAS-gars^P234KY_1^* were previously shown to be expressed at equivalent levels and both proteins localized to the cytoplasm within the neuronal populations studied (18). Here, we first analysed the localization of the GlyRS proteins expressed from our new *UAS-gars^WT_2^* (Fig. 1A) and *UAS-gars^P234KY_2^* (Fig. 1B) constructs, when driven ubiquitously (*1032-GAL4*). Both GlyRS^WT^ and GlyRS^P234KY^ localized in a similar cytoplasmic diffuse pattern in the cell bodies of larval central nervous system neurons (Fig. 1A and B). Both proteins were also observed in the axonal bundles that contain afferent motor and sensory neurons ensheathed in glia (Fig. 1A and B), as well as the neuropil region of axons and dendrites, which was labelled with bruchpilot (Brp, Fig. 1A and B). We also found analogous localization patterns when *UAS-gars^WT_1^* and *UAS-gars^P234KY_1^* were used (data not shown), as previously described (18). As GlyRS was observed in the axonal bundles, we expressed both GlyRS^WT^ and GlyRS^P234KY^ using a neuronal-specific driver (*elav-GAL4*) to determine whether GlyRS reaches the most distal region of the motor neuron, the NMJ (Fig. 1C and D). Both GlyRS^WT^ and GlyRS^P234KY^ did not become enriched at the larval NMJ, suggesting that neuronally expressed GlyRS does not reach the peripheral synapses of the neuromuscular system.

We next looked to identify any toxic effects of wild-type or mutant GlyRS. To this end, we analysed lethality and motor function deterioration, both of which are common features of *Drosophila* models of neurodegenerative diseases (21–23). Previous experiments have shown that ubiquitous expression of *gars^P234KY^* leads to very early lethality, whereas neuronal expression causes late pupal lethality (18). This suggests that, in addition to neuronal involvement, toxicity may manifest or be contributed from tissues beyond the nervous system. We therefore analysed tissue-specific lethality and motor function deterioration (Fig. 1E–I). To take advantage of temperature-dependent GAL4 activity (24) and thus express transgenes at different levels, we performed experiments at different temperatures; we cultured flies at both 20°C (Fig. 1E) and 25°C (Fig. 1F), which correspond to low and high transgene expression levels, respectively. Concurrent with the previous study, we demonstrated that ubiquitous expression of *gars^P234KY_1^* and *gars^P234KY_2^*, using the moderately expressing *1032-GAL4* driver, leads to early lethality at both 20°C (Fig. 1E) and 25°C (Fig. 1F), with the majority of flies dying during the pupal stages (designated White Pupa and Pupa). Ubiquitous expression of both wild-type constructs (*UAS-gars^WT_1^* and *UAS-gars^WT_2^*) had no overt affect when compared with control (*1032-GAL4; UAS-CD8::GFP*), at either temperature (Fig. 1E and F). Tissue-specific GlyRS^P234KY^ toxicity was then analysed by expressing *gars^P234KY^* constructs in the mesoderm (*how^24B^-GAL4,* called *how-GAL4* henceforth), muscle (*MHC-GAL4*) or neurons (*elav-GAL4*). Mesodermal and muscle expression of either *gars^P234KY^* construct reduced survival, with mesodermal expression leading to earlier lethality than muscle at both temperatures. Surprisingly, given the previous study (18), *gars^P234KY^* expression in the nervous system caused only a modest affect at 20°C, whereas leading to more pronounced lethality at 25°C (Fig. 1F). This result was confirmed using both versions on the *UAS-gars^P234KY^* transgene. This discrepancy is likely due to the different expression levels of the GAL4 promoters used in the two studies.

A number of flies ubiquitously expressing *gars^P234KY^* that reached the pupal stage at 25°C failed to eclose from the pupal case (Fig. 1G). In addition, the few successfully eclosed flies failed to expand their wings, mirroring defects that have been previously observed in classical neurodegenerative models that display axonal transport and neuronal viability deficits (25,26).

We next looked at how the P234KY mutation affects larval motor function by assessing the peristaltic waves of muscle contraction (Fig. 1H and I). When larvae were reared at 20°C, reduced muscle contractions were detected upon ubiquitous, mesodermal and muscle expression of both GlyRS^P234KY_1^ and GlyRS^P234KY_2^ (Fig. 1H). At 25°C, when transgenes were expressed at higher levels, muscle contraction defects were also now observed upon neuronal expression of *gars^P234KY^*, although not to the same extent as defects caused by mesodermal and muscle expression (Fig. 1I). These results suggest that multiple tissues may be involved in the selective neuropathy caused by mutant GlyRS.

To confirm the specificity of the toxicity to mutant *gars*, we tested an additional, CMT2D-associated GlyRS mutant in the muscular system. Importantly, the second *gars* mutation, *G240R*, was shown to exert the same pathological effects as *P234KY* when expressed ubiquitously, in the mesoderm, or in muscle (Supplementary Material, Fig. S1A).

These findings reveal a clear dominant, toxic gain-of-function for mutant, but not wild-type, GlyRS that is mediated, in part, through tissues beyond the nervous system. Moreover, the severity of pathology was modulated by temperature, suggesting the importance of mutant GlyRS levels. In this study, we now aim to characterize these non-cell autonomous defects to understand how mutant *gars* expressed in non-neuronal cell types may contribute to the selective deterioration of the nervous system.

### GlyRS^P234KY^ larvae display non-cell autonomous NMJ growth defects

**Figure 2.**
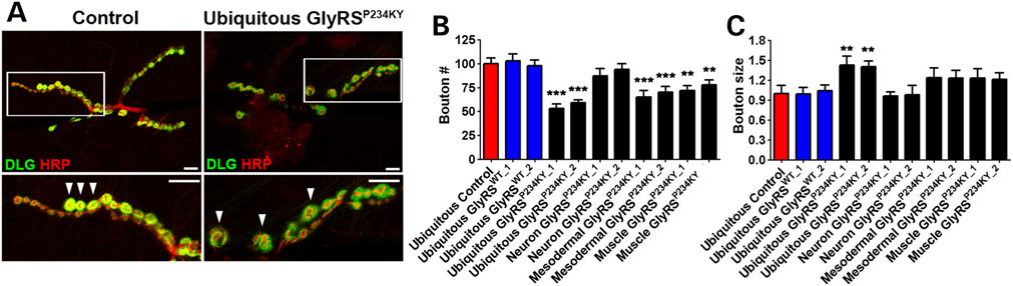
*gars^P234KY^* expression causes NMJ defects. NMJs consist of boutons that grow in lines resembling beads on a string (arrowheads, **A**). Nerves were visualized with anti-HRP (red) and the post-synaptic apparatus with anti-DLG (green). (A) Representative NMJs from muscles 6 and 7 of control (*1032-GAL4*) and ubiquitous *gars^P234KY^* (*1032-GAL4; UAS-gars^P234KY^*) larvae at the late L3 stage. (**B**) Ubiquitous, mesodermal (*how-GAL4*) and muscle (*MHC-GAL4*) expression of *gars^P234KY^* caused a reduction in bouton number. (**C**) Ubiquitous, mesodermal and muscle expression also lead to an increase in bouton size. Neuronal *gars^P234KY^* expression (*elav-GAL4*) caused no overt synaptic phenotype. For all graphs, *P < 0.05, **P < 0.01, ***P < 0.001 Bonferroni's/Dunn's multiple comparison test. At least 20 larvae were scored per NMJ. Scale bars = 10 µm.

As motor defects were present at larval stages in mutant flies, we looked for changes in the structure and innervation pattern of the larval NMJ. All subsequent experiments were performed at 25°C, as significant toxicity is observed at this temperature in all the tissues studied. Abdominal ventrolateral muscles 6 and 7 are innervated in a stereotyped manner in *Drosophila* larvae by a defasciculated segmental nerve d. Wild-type NMJs grow by adding circular synaptic specializations called boutons, which form structures like beads on a string, where both pre- and post-synaptic regions closely overlap (Fig. 2A). Larvae ubiquitously expressing *gars^P234KY^* displayed reduced bouton number and increased bouton size when compared with wild-type and *gars^WT^*-overexpressing flies (Fig. 2B and C). No gross changes in muscle size or structure were observed in any of the genotypes analysed (Supplementary Material, Fig. S2). Again, tissue-specific GlyRS^P234KY^ toxicity was analysed by expressing *gars^P234KY^* in mesoderm (*how-GAL4*), muscle (*MHC-GAL4*) or neurons (*elav-GAL4*), whereas flies ubiquitously expressing *gars^WT^* were used as controls. Bouton number defects were also observed in larvae with mesoderm and muscle-driven GlyRS^P234KY^, whereas neuronal *gars^P234KY^* expression caused no overt synaptic phenotype (Fig. 2B and C).

### Muscle *gars^P234KY^* expression causes early denervation defects

**Figure 3.**
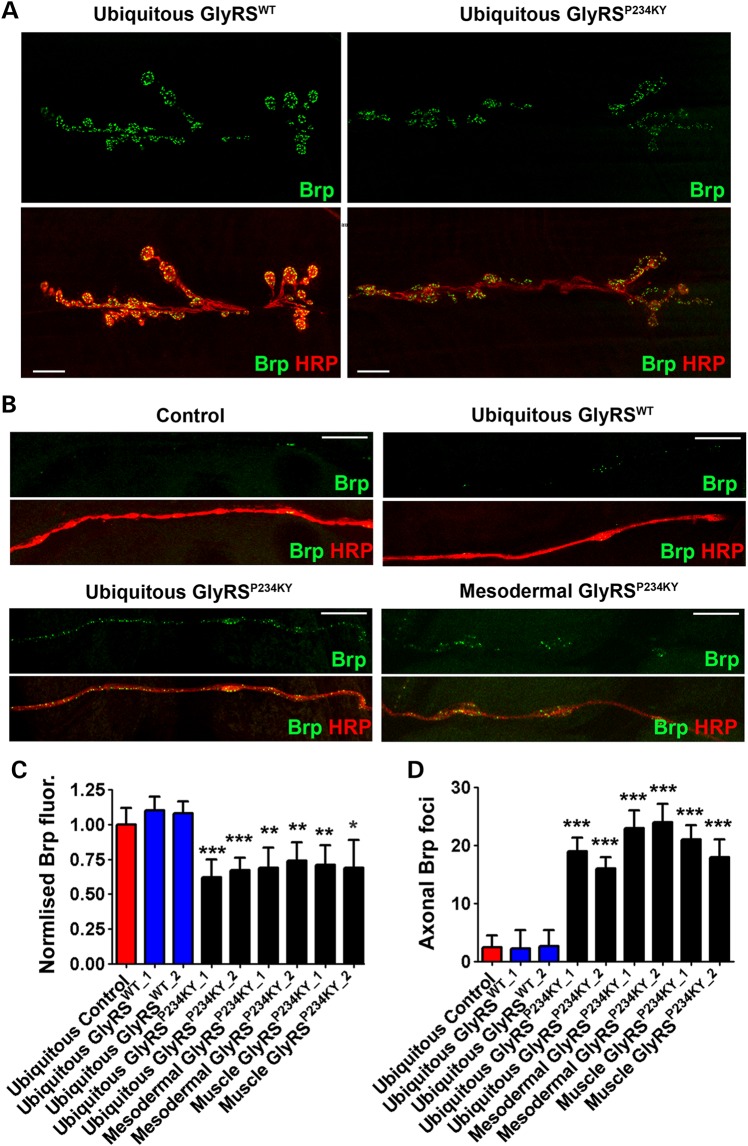
*gars^P234KY^* larvae display NMJ denervation and axonal defects. (**A**) Representative NMJs from late L3 larvae ubiquitously (*1032-GAL4*) expressing *gars^WT^* and *gars^P234KY^*. (**B**) Representative axonal segment pictures taken of the transverse nerve (TN) over muscles 6 and 7 in abdominal segment A2 in late L3 control, *gars^WT^* (*1032-GAL4*), *gars^P234KY^* (*1032-GAL4*) and *gars^P234KY^* mesodermal (*how-GAL4*) larvae. (**C**) Quantification of the normalized Brp fluorescence at the NMJ showing ubiquitous, mesodermal and muscle *gars^P234KY^* expression leads to reduced Brp signal. (**D**) Quantification of the number of Brp-positive foci in the TN showing that ubiquitous, mesodermal and muscle *gars^P234KY^* expression leads to increased Brp accumulation. For all graphs, *P < 0.05, **P < 0.01, ***P < 0.001 Bonferroni's/Dunn's multiple comparison test. At least 20 NMJs and axons were analysed per genotype. Scale bars = 10 µm.

As the synaptic structure was affected in *gars* mutant flies, we next looked to see if axonal and synaptic degenerative defects presented at the late L3 larval stage. CMT2D patients display muscle weakness and axonal degeneration (27). In *Drosophila*, denervation of the synapse can be analysed by detecting the loss or reduction of pre-synaptic antigens such as the active zone protein Brp (28). In ubiquitous, mesodermal and muscle *gars^P234KY_2^*-expressing larvae, we found synaptic Brp intensity was reduced compared with control (Fig. 3A and C). This was not seen when *gars^WT^* was overexpressed (Fig. 3A and C). It is worth noting that HRP immunoreactivity was reduced at the synapse in ubiquitous and muscle *gars^P234KY^*-expressing larvae, suggesting that the defects were due to a degeneration of the pre-synapse and not the mis-regulation of Brp anchoring. In addition to the synaptic denervation, Brp was found to accumulate along the axon (Fig. 3B and D), which is a potential indicator of neurodegeneration (29) or axonal transport defects (30). Together, these findings demonstrate that synaptic and axonal defects can be caused by a non-cell autonomous mechanism.

### GlyRS^P234KY^ accumulates at the synapse

**Figure 4.**
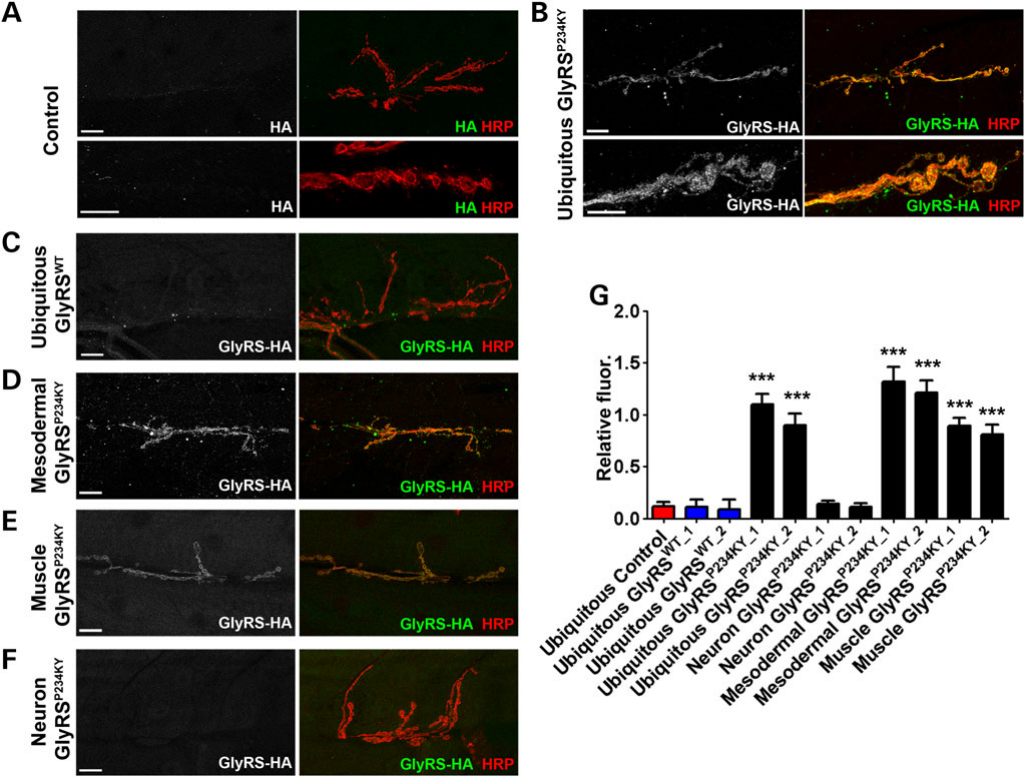
GlyRS^P234KY^ secretion leads to non-cell autonomous build-up at the neuronal membrane. (**A**–**F**) L3 larval NMJs were stained under non-permeabilization conditions to assess GlyRS^P234KY^ binding to the neuronal extracellular membrane. (A) HA staining was not observed at the membrane in control flies. (B) Ubiquitous *gars^P234KY^* expression, but not (C) *gars^WT^* expression, leads to a build-up of GlyRS at the neuronal membrane. (D) Mesodermal and (E) muscle expression of *gars^P234KY^* also causes a build-up of GlyRS^P234KY^ on the neuronal membrane. GlyRS^P234KY^ appeared to associate with the whole pre-synapse and axonal regions. (F) Neuronally expressed GlyRS^P234KY^ did not localize to the synapse. (**G**) Quantification of the HA fluorescence at the synapse. Ten NMJs were analysed per genotype. Scale bars = 10 µm.

As both developmental and degenerative processes appear to affect the mutant *gars* synapse, we next analysed the localization of GlyRS^P234KY^. As the canonical function of GlyRS is strictly intracellular, but there appears to be a non-cell autonomous component to pathology in our model, we studied the extracellular localization of GlyRS^P234KY^ using non-permeabilization conditions (without detergent) (Fig. 4). The relative florescence of GlyRS at the synapse was measured and normalized to the background intensity (Fig. 4G). When *gars^P234KY^* was ubiquitously expressed, GlyRS^P234KY^ was found to broadly cover the neuromuscular synapse in puncta that were mainly closely associated with the HRP-labelled pre-synaptic membrane (Fig. 4A and B). Signal was not detectable in controls or when *gars^WT^* was expressed (Fig. 4C). This was again seen when GlyRS^P234KY^ was driven independently in the mesoderm (Fig. 4D) or in the muscle (Fig. 4E). These experiments suggest that GlyRS^P234KY^, but not GlyRS^WT^, is secreted into the extracellular milieu at the NMJ and is able to localize to the outside of the pre-synaptic membrane. In addition, neuron-expressed GlyRS^P234KY^ (*elav-GAL4*) did not appear at the pre-synapse (Fig. 4F), suggesting that the synaptic build-up of mutant GlyRS is due to a non-cell autonomous mechanism. Ubiquitous, mesodermal and muscle-expressed mutant GlyRS localized to the NMJ when using both *UAS-gars^P234KY_1^* and *UAS-gars^P234KY_2^* constructs (Fig. 4G), whereas muscle-expressed GlyRS^WT^ did not (Supplementary Material, Fig. S1B).

### GlyRS toxicity and synapse binding, but not secretion, are dependent on the WHEP domain

**Figure 5.**
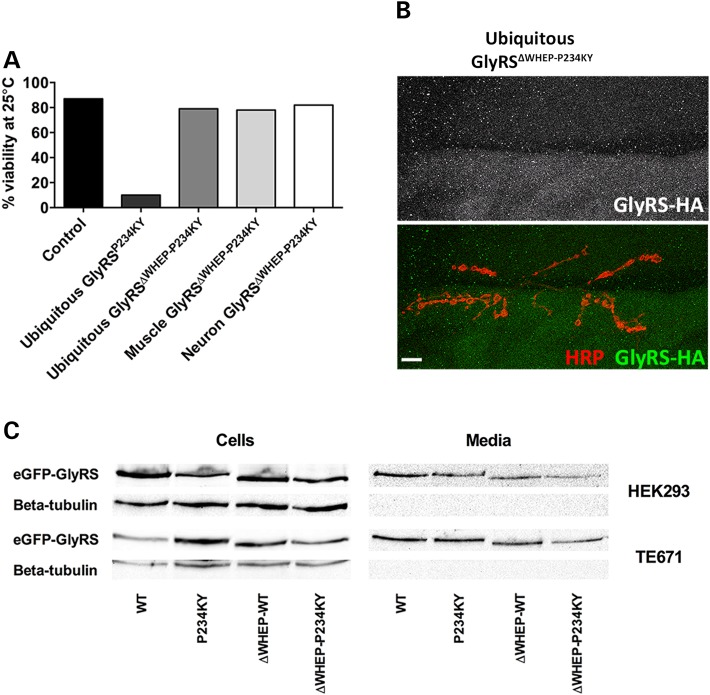
GlyRS toxicity and synapse binding, but not secretion, are dependent on the WHEP domain. (**A**) Removal of the WHEP domain abrogated the pathology associated with both ubiquitous and muscle expression of mutant *gars in vivo*; *gars^ΔWHEP-P234KY^* expression had no effect on larval viability. (**B**) GlyRS^ΔWHEP-P234KY^ did not associate with the neuronal membrane (using non-permeabilization conditions). (**C**) To determine whether WHEP deletion restricts GlyRS secretion and is the principal cause of the abolished mutant pathology, eGFP-tagged *GARS^WT^* and *GARS^P234KY^* with or without the WHEP domain were expressed in human kidney (HEK293) and muscle-like (TE671) cells. Deletion of the WHEP domain did not affect secretion of wild-type or mutant GlyRS. The localization of all GlyRS proteins was similar in both cell lines and with each protein tag (data not shown). Scale bars = 10 µm.

GlyRS was recently shown to be secreted by macrophages and to be present in human and mouse serum, serving as a defence against ERK-activated tumour formation (31). Several other ARS proteins are also known to be secreted (32). The capacity of mesoderm- and muscle-expressed GlyRS^P234KY^, through secretion, to exert non-cell autonomous effects on the nervous system appears to be an integral component to the pathology we have observed. A candidate determinant for GlyRS secretion is the N-terminal WHEP domain, which currently has no known function in GlyRS and is dispensable for aminoacylation activity (20). We therefore investigated the importance of the WHEP domain for GlyRS toxicity by generating a WHEP-deletion fly. Intriguingly, removal of the WHEP domain from GlyRS^P234KY^ (GlyRS^ΔWHEP-P234KY^) abrogated the fly viability (Fig. 5A) and NMJ defects (data not shown) caused by GlyRS^P234KY^ (Fig. 5A). Furthermore, build-up of GlyRS^ΔWHEP-P234KY^ was not found at the NMJ, suggesting that synaptic mutant GlyRS accumulation is integral to pathology (Fig. 5B). To show that our transgenes were successfully expressed and to assess protein localization, we used *UAS* ‘flip-out’ clones expressing *UAS-gars^P234KY^* and *UAS-gars*^Δ*WHEP-P234KY*^. Both GlyRS^P234KY^ and GlyRS^ΔWHEP-P234KY^ were expressed and localized in the cytoplasm of disc clones (Supplementary Material, Fig. S3A), indicating that deletion of the WHEP domain is unlikely to affect protein stability. This is corroborated by previous observations that deletion of the WHEP domain does not affect the canonical function of GlyRS and does not prevent the crystalization of the truncated protein (20). Finally, to determine whether WHEP deletion restricts GlyRS secretion and is the principal cause of the abolished mutant pathology, we expressed *GARS^WT^* and *GARS^P234KY^* with and without the WHEP domain in human kidney (HEK293) and muscle-like (TE671) cells. Contrary to our starting hypothesis that the WHEP domain may be a determinant of secretion, we found that its deletion did not affect secretion of eGFP- or V5-tagged versions of the protein (Fig. 5C, Supplementary Material, Fig. S4A). To confirm that the secretion of GlyRS proteins was not simply a product of the overexpression system, we demonstrated that eGFP-tagged survival motor neuron (Smn) protein is not secreted under similar conditions in both HEK293 (Supplementary Material, Fig. S4B) and TE671 cells (data not shown). The higher expression of Smn-eGFP than GlyRS-eGFP makes this result all the more convincing. Smn was chosen as a control, because, like GlyRS, it is expressed both in the nucleus and cytoplasm (33), it has been identified in axonal projections (10,34), and its dysfunction is associated with a disorder that affects the lower motor neurons (spinal muscular atrophy) (34,35). The similar subcellular localization patterns of eGFP-tagged GlyRS and Smn were confirmed by immunofluorescence (Supplementary Material, Fig. S4C). Altogether, this work indicates that the GlyRS WHEP domain, although not required for secretion, nevertheless is integral to pre-synaptic accumulation of muscle-expressed mutant GlyRS.

## Discussion

This work reveals a novel mechanism for the pathology associated with the peripheral neuropathy CMT2D, and identifies a complex series of events leading to the characteristic selective neuromuscular toxicity. CMT2D, like many other neuromuscular diseases, is caused by mutations in a widely and constitutively required gene, yet pathology is strictly limited to the neuromuscular system. Despite having identified the disease-causing genes for many motor neuropathies, there remains a dearth of information on the molecular mechanisms underlying selective motor neuron vulnerability (36,37). Here, we show two unexpected acquired functions of mutant GlyRS in a *Drosophila* model, wherein ubiquitous expression of mutant *gars* leads to specific, progressive NMJ degeneration. First, we show that pathology has a non-cell autonomous contribution—mutant GlyRS exerts significant neuronal defects when expressed just in muscle (Figs 1–4). This non-neuronally derived mutant GlyRS causes a loss of motility (Fig. 1H and I), synaptic degeneration (Figs 2B, C and 3A, C), and, ultimately, a reduction in lifespan (Fig. 1E and F). Secondly, we find mutant GlyRS associated with the neuronal membrane at the synapse when expressed ubiquitously or in muscle, but not when expressed in the neuron (Fig. 4). Wild-type GlyRS was not observed to accumulate at the NMJ; however, we show that both wild-type and mutant GlyRS proteins are secreted (Fig. 5). This suggests that CMT2D-associated mutations in GlyRS, including P234KY, may cause the selective neuropathy by either (i) a toxic gain of neuronal binding function or (ii) a notable increase in neuronal binding affinity. It has been shown that tRNA synthetases, including GlyRS, are secreted and have non-canonical functions (12,31). For example, GlyRS is secreted by macrophages in response to serum-circulating FAS ligand (31), whereas tryptophanyl-tRNA synthetase (WARS) is secreted by vascular endothelial cells (12). It is worth noting that muscle cells are required to secrete a number of factors to maintain synapse integrity and growth (38,39). It is therefore possible that wild-type GlyRS possesses a second non-canonical function specific to the NMJ that is dependent upon sub-synaptic secretion.

Mutant *gars* expression leads to synaptic maturation abnormalities that present as decreased bouton number and increased bouton size (Fig. 2). Although not yet observed in patients, NMJ maturation defects precede lower motor neuron degeneration in *Gars* mice (13), indicating that defective synapse development may play an important role in pathology. In addition to the synaptic developmental defects, we observed a potential deficit in axonal transport (Fig. 3B and D). Although not yet assessed in CMT2D, axonal transport defects have been reported in a range of CMT2 models (10,35,40–42) and may play a part in numerous other disorders affecting the peripheral nerves (43–48). We show that mutant *gars* expression causes regions of the synapse to degenerate at late larval stages, as identified by parts of the NMJ lacking post-synaptic anchoring (Figs 2 and 3).

Our study demonstrates a potential role for non-cell autonomous mechanisms in CMT2D, a topic that has recently gathered interest in a number of other nervous system disorders, including the motor neuron disease amyotrophic lateral sclerosis (11). Previous work has shown that mutant GlyRS causes cell autonomous neuronal pathology in a *Drosophila* model (18). In that study, overexpression of mutant *gars* exclusively in neurons resulted in both electrophysiological and morphological defects. It is likely that both neuronal and non-neuronal mechanisms contribute to CMT2D neuropathology; and it will therefore be of great interest to analyse the interaction between these modular pathological mechanisms in future studies.

Throughout evolution, ARSs have acquired domains and motifs that are not required for the canonical aminoacylation function of the proteins (49). One such domain is the helix–turn–helix motif known as the WHEP domain, which has been shown to be involved in several non-canonical ARS functions (50,51). For example, removal of the WARS WHEP domain promotes angiostatic growth through interactions with VE-cadherin (51), whereas a Glu–Pro dual activity tRNA synthetase (52) translationally represses a number of mRNAs by WHEP domain-mediated binding to transcript 3′UTRs (20,24). We show that removal of the WHEP domain from GlyRS^P234KY^ prevents the build-up of mutant protein at the NMJ and abrogates the longevity defects (Fig. 5A and B), suggesting that the WHEP domain is required for synaptic mutant GlyRS accumulation and toxicity. As WHEP domain removal did not affect secretion *in vitro* (Fig. 5C, Supplementary Material, Fig. S4), this suggests that the domain perhaps determines binding affinity of mutant GlyRS to the pre-synapse.

In summary, we show that, mediated by the WHEP domain, mutant GlyRS escapes muscle cells to accumulate at the pre-synaptic membrane, before internalization into ectopic regions of the motor neuron. In parallel, we see synaptic maturation defects that ultimately lead to NMJ degeneration, similar to what is observed in mice (13), and progressive neuromuscular and survival defects. Our findings provide novel insight into the selective effect on the neuromuscular system of mutations in a widely expressed protein.

## Materials and Methods

### General methods

Reagents were obtained from Sigma-Aldrich unless otherwise stated. All *Drosophila* stocks were cultured on standard molasses/maize meal and agar medium in plastic vials or bottles at 25°C. Coding sequences of *D. gars^WT^*, *gars^P234KY^*, *gars^G240R^* and *gars^ΔWHEP-P234KY^* were amplified using primers containing KpnI sites, sub-cloned into pUAST and injected into embryos. We wish to thank the following for stocks/reagents: *1032-GAL4*, *Act5C-GAL4*, *elav-GAL4* and *how-GAL4* (BDSC, Indiana University), *BG487-GAL4* and *C57-GAL4* (Vivian Budnik), *nrv2-GAL4* (Paul Salvaterra), *69B-GAL4* (Clive Wilson), and *UAS-gars^WT_1^* and *UAS-gars^P234KY_1^* (Albena Jordanova).

### Drosophila *behavioural* assays

Adult viability assays were conducted by crossing *GAL4* driver stocks to lines harbouring *gars* overexpression or to *w^1118^* for controls. First instar larvae for each genotype were counted and placed on apple juice plates with yeast, and the development of the larvae was noted (53). Fresh yeast was added daily. Survivors were counted each day. Twenty-five flies per genotype were scored over four independent experiments. Measurement of motor function involved placing individual age-matched larvae at the centre of a 0.7% (w/v) agar plate and counting the forward body wall contractions exhibited in 2 min. At least 15 larvae per genotype were scored for muscle contraction assays.

### Drosophila *larval* NMJ analysis

Larvae were reared and dissected, as previously described (54), and at least 20 NMJs (muscles 6–7, hemisegment A2) per genotype per stage were scored (55). For immunohistochemistry, anti-discs large (DLG, DSHB, 4F3), anti-HRP (Jackson Immuno, 323-005-021), anti-bruchpilot (DSHB, nc82) and anti-HA (Santa Cruz, sc-805) were all used at 1/100, with AlexaFluor 488 goat anti-rabbit and AlexaFluor 633 goat anti-mouse (Invitrogen) secondary antibodies at 1/1000. Z-stacks were taken using a Leica SP5 laser confocal microscope and analyses performed using ImageJ and Photoshop (Adobe). For BRP analysis in the axon, optical sections of 0.2 µm were taken using a laser-scanning confocal microscope (Leica TCS SP5 II confocal microscope). Bouton size analysis was performed by averaging two perpendicular diameter measurements of individual Ib boutons. All observable Ib boutons, which were identified by intense DLG staining, were measured per NMJ. All digital analyses and fluorescence measurements were performed using ImageJ. For BRP and HA staining at the synapse, average fluorescence intensity was analysed over the whole synapse (marked by HRP staining), again using optical sections of 0.2 µm. For HA staining, this was normalized to the background fluorescence intensity in the muscle.

### Clonal analysis

All *UAS* ‘flip-out’ clones were generated using the inducible driver: *HsFLP*, *UAS-GFP_nls_*; *UAS-Dcr2*; *tub>GAL80>GAL4/SM5, Cyo-TM6, Tb.* Virgin females of this genotype were crossed to males with *UAS-shRNA* or *UAS-ORFs* for clonal knockdown or overexpression, respectively. Progeny were heat-shocked at 24 h for imaginal tissue. Larvae were aged until third instar and non-TM6B (non-tubby) animals were selected. *GAL4*-expressing clones were identified by the presence of nuclear GFP marker.

### Cell culture

Human HEK293 (human embryonic kidney) and TE671 (muscle-like) cells were maintained in Dulbecco's modified Eagle's medium (DMEM) with 4.5 g/l d-glucose and pyruvate (Invitrogen) supplemented with 10% (v/v) heat-inactivated fetal bovine serum (Invitrogen). Cells were kept at 37°C in a 5% (v/v) CO_2_ humidified atmosphere and sub-cultured every 4–6 days when ≈70–90% confluent.

### Cell secretion assays and western blotting

HEK293 cells were plated at 2.5 × 10^5^ cells per well of a 24-well plate on poly-d-lysine (BD Biosciences), and TE671 cells were plated at 1.0 × 10^5^ on Geltrex (Invitrogen). 6 ± 0.5 h post-plating, HEK293 cells were transfected with 500 ng DNA using 1 μl Lipofectamine 2000 Transfection Reagent (Invitrogen), as previously described (43). TE671 cells were transfected/magnetofected with 1 μg DNA using a combination of 1 μl Lipofectamine 2000 and 0.5 μl NeuroMag beads (Oz Biosciences), with an initial incubation step of 20 min at 37°C on a magnetic plate (Oz Biosciences). At 15–18 h later, cell media was discarded and the cells carefully washed twice with 0.2 ml pre-warmed phosphate-buffered saline. Cells were then incubated at 37°C for 6 h in 0.5 ml serum-free DMEM. The media from 4–6 wells/condition were spun at 1500 × *g* for 4 min and the supernatant re-spun at 4000 × *g* for 10 min. The media were then concentrated using VIVAspin columns (MWCO 10 kDa, Sartorius) at 4000 × *g* for 18–28 min. A 1.5% (v/v) protease inhibitor cocktail was added to the concentrated media before cell protein extraction and western blotting, as previously described (43). The following antibodies were used for western blotting: anti-β-actin (Cell Signaling Technology, 8H10D10, 1/5000), anti-β-tubulin (Abcam, ab15568, 1/5000), anti-HA (Roche, 1-867-423, 1/1000), anti-GFP (Abcam, ab290, 1/5000) and anti-V5 (Invitrogen, R960-25, 1/5000). Cells were stained with anti-GFP at 1/1000 as previously described (43).

### DNA vector creation

The cytoplasmic isoform of human *GARS* was cloned into the pcDNA6.2/C-EmGFP-DEST and pcDNA6.2/V5-DEST vectors (Invitrogen) to express GlyRS with a C-terminal eGFP or V5 tag, respectively. Smn with a C-terminal eGFP tag was expressed from pEGFP-N1 (43). To create the *GARS^P234KY^* and ΔWHEP (residues 71–126) mutation constructs, a Q5 Site-Directed Mutagenesis Kit (New England Biolabs) was used, as per the manufacturer's instructions, with the following primers: WT-P234KY_F 5′-TTT CAT TGG GAA ATA TGG AGG AAA CAT GC-3′, WT-P234KY_R 5′-GTC TTG AAC ATT AAG TTA AAA GAC-3′, ddWHEP_F 5′-ACC CTG AAG AGG AGG TTT TTC-3′ and ddWHEP_R 5′-TGC TAG CCT CAG AGG TGC-3′.

### Statistical analysis

When normally distributed, datasets were statistically analysed using either an unpaired *t*-test with Welch's correction or a one-way analysis of variance with Bonferroni's multiple comparison test. If the data did not pass normality testing, the non-parametric Mann–Whitney *U* test or Kruskal–Wallis test with Dunn's multiple comparison test was used. GraphPad Prism 5 software was used for all statistical analyses. Means + SEM are plotted for all graphs unless otherwise stated.

## Supplementary Material

Supplementary Material is available at *HMG* online.

## Funding

This work was supported by the Medical Research Council (S.J.G., M.Z.C.), the Oxford Biomedical Research Centre (M.Z.C.), the French Muscular Dystrophy Association (AFM-Telethon) (J.N.S., K.T., M.Z.C.), the Wellcome Trust (J.N.S.) and the National Institutes of Health (NS054154 to R.W.B.). Funding to pay the Open Access publication charges for this article was provided by the Wellcome Trust.

## Supplementary Material

Supplementary Data
