# Supplementary material for: Dominant, toxic gain-of-function mutations in gars lead to non-cell autonomous neuropathology
Source: Hum Mol Genet. 2015 May 13;24(15):4397–406. doi: 10.1093/hmg/ddv176 (PMC4492401; doi:10.1093/hmg/ddv176)
Supplement: Supplementary Data [file supp_24_15_4397__index.html]

Dominant, toxic gain-of-function mutations in gars lead to non-cell autonomous neuropathology — Dominant, toxic gain-of-function mutations in gars lead to non-cell autonomous neuropathology — Dominant, toxic gain-of-function mutations in gars lead to non-cell autonomous neuropathology — Supplementary Data 

# Dominant, toxic gain-of-function mutations in *gars* lead to non-cell autonomous neuropathology

## Supplementary Data

Supplementary Data

- Supplementary Data - Pdf file
