# Supplementary material for: Dominant, toxic gain-of-function mutations in gars lead to non-cell autonomous neuropathology
Source: Hum Mol Genet. 2015 May 13;24(15):4397–406. doi: 10.1093/hmg/ddv176 (PMC4492401; doi:10.1093/hmg/ddv176)
Supplement: Supplementary Data [file supp_ddv176_ddv176supp.pdf]

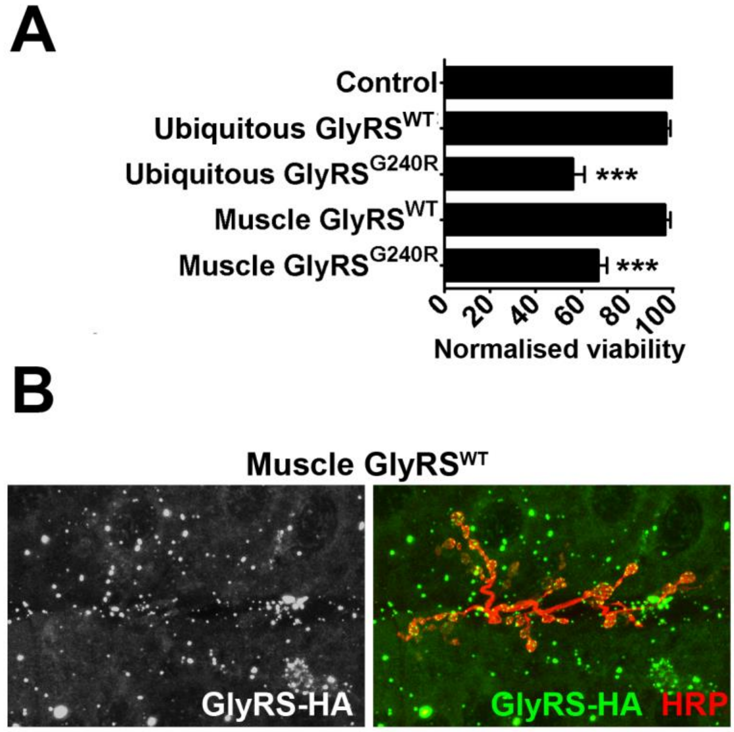

Figure S1. Pathological effects of mutant GlyRS are replicated by a second *gars* mutation. **(A)** Ubiquitous and mesodermal expression of a second, CMT2D-associated *gars* mutation, G240R (*1032-GAL4; UAS-gars*^{G240R}), causes a reduction in adult viability comparable to P234KY. **(B)** A high gain confocal image of *gars*^{WT} muscle expression (MHC-GAL4) showing no enrichment at the NMJ.

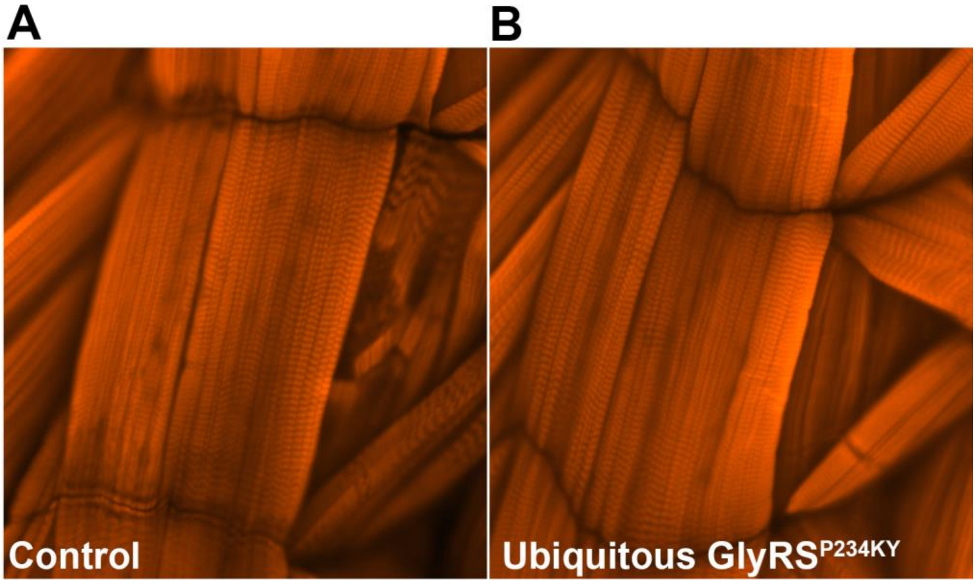

Figure S2. Ubiquitous *gars*^{P234KY} expression causes no gross muscle structure defects. Phalloidin-stained muscle preparations of control (**A**, *1032-GAL4)* and ubiquitous GlyRS^{P234KY} (**B**, *1032-GAL4; UAS-gars*^{P234KY}) larvae at the late L3 stage showing no overt muscle structure defects.

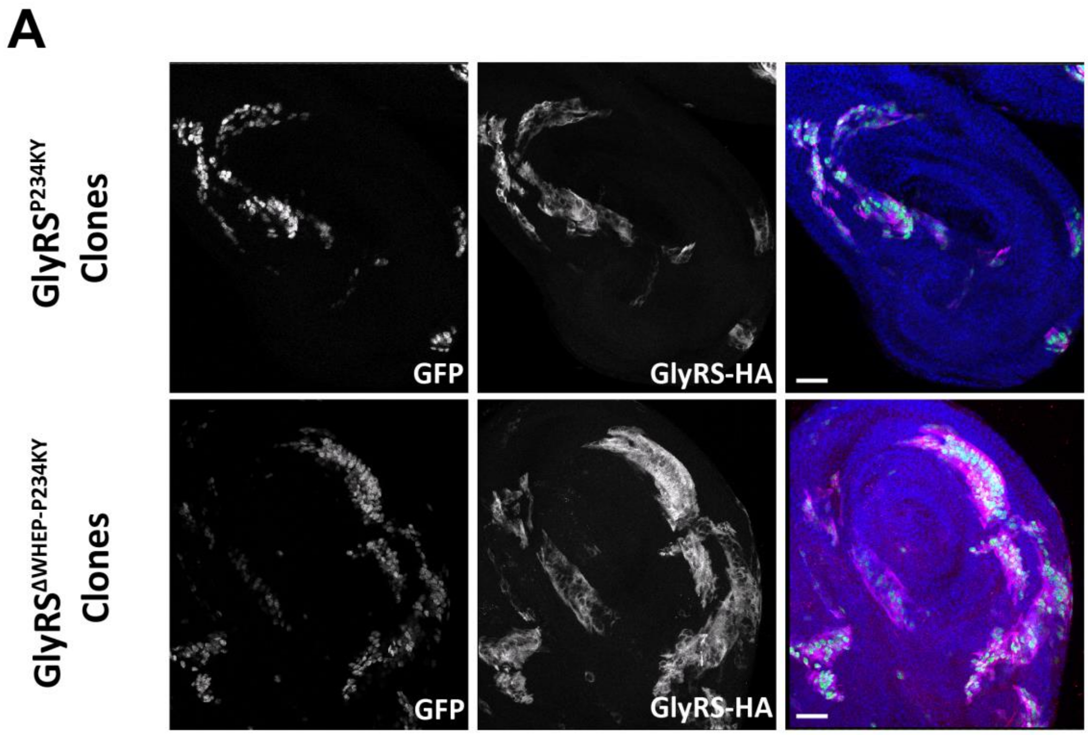

Figure S3. Deletion of the WHEP domain does not disturb *gars* expression. UAS "flip-out" clones expressing *UAS-gars*^{P234KY} and *UAS-gars*$^{\Delta WHEP\text{-}P234KY}$ were generated using an inducible driver that allows for GFP-labeled clone formation (*HsFLP, UAS-GFPnls; UAS-Dcr2; tub>GAL80>GAL4 / SM5, Cyo-TM6, Tb*). Both GlyRS^{P234KY} (top panels) and GlyRS$^{\Delta WHEP\text{-}P234KY}$ (bottom panels) were expressed and localised in the cytoplasm of disc clones. Scale bars = 10 µm

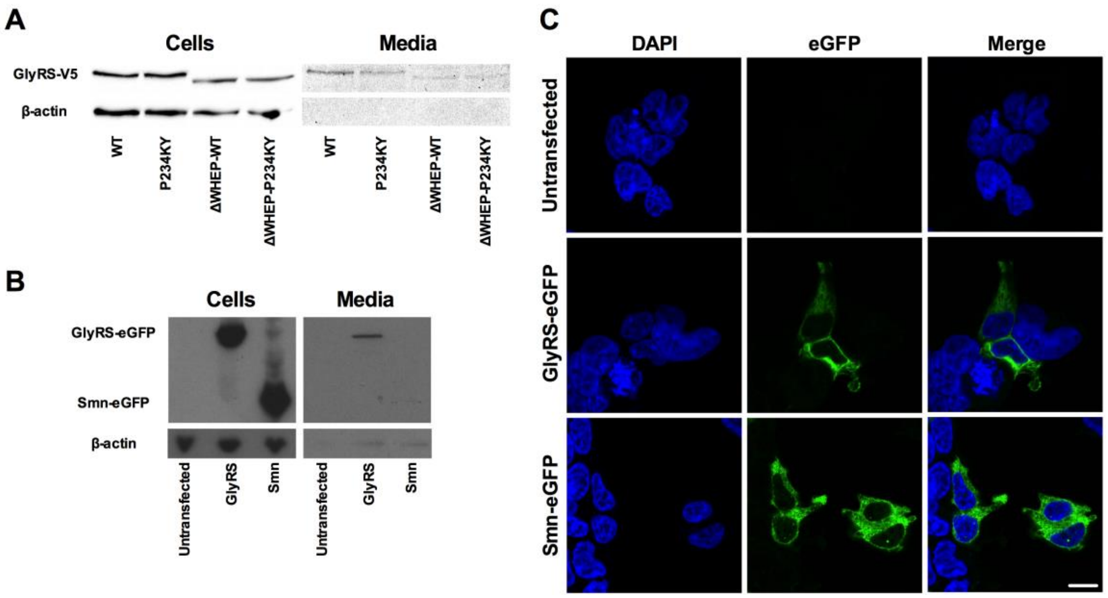

Figure S4. GlyRS secretion is independent of the C-terminal tag and is a feature specific to the protein. (A) V5-tagged GlyRS^{WT} and GlyRS^{P234KY} with and without the WHEP domain are all secreted at similar levels into the media from human kidney (HEK293) cells. **(B)** Transfected Smn-eGFP was not secreted into HEK293 cell media, suggesting that GlyRS is indeed specifically secreted rather than the result being a non-specific effect of exogenous protein expression. Note the greater level of Smn-eGFP expression than GlyRS-eGFP. This result was also confirmed in TE671 cells (data not shown). **(C)** GlyRS-eGFP and Smn-eGFP display similar localisation patterns in transfected HEK293 cells (also confirmed in TE671 cells, but not shown), indicating that Smn is a worthy control to test for the specificity of secretion to GlyRS protein. Scale bar = 10 µm
